# Case report: Fatal hematogenous gastrosplenic mucormycosis in an immunocompetent adult following sigmoid perforation, septic shock, and short-term stress glucocorticoids

**DOI:** 10.3389/ffunb.2026.1940494

**Published:** 2026-09-29

**Authors:** Jiewei Liu, Wei Zhang, Hua Chen

**Affiliations:** 1 Department of Emergency Medicine, Fuzong Clinical Medical College of Fujian Medical University, Fuzhou, Fujian, China; 2 Department of Emergency, 900th Hospital of PLA Joint Logistic Support Force, Fuzhou, Fujian, China; 3 Department of Emergency Medicine, Fuzong Teaching Hospital of Fujian University of Traditional Chinese Medicine (900th Hospital), Fuzhou, Fujian, China

**Keywords:** gastric perforation, liposomal amphotericin B, *Mucorales*, Mucormycosis, sigmoid colon perforation, splenic infarction

## Abstract

**Background:**

Mucormycosis is an aggressive angioinvasive fungal infection that predominantly affects chronically immunosuppressed individuals. Hematogenous dissemination of *Mucorales* causing synchronous gastric full-thickness perforation and total splenic infarction is exceptionally rare, particularly in immunocompetent hosts without chronic comorbidities. To our knowledge, this presentation has not been reported in a patient with only three days of stress-dose glucocorticoid therapy following sigmoid perforation and septic shock.

**Case presentation:**

We report a fatal case of synchronous gastric perforation and total splenic infarction secondary to hematogenous *Mucorales* dissemination in a previously healthy adult who developed septic shock from spontaneous sigmoid perforation. The patient received only three days of guideline-recommended stress-dose hydrocortisone and had no pre-existing diabetes, malignancy, or organ transplantation. Routine serum 1,3-β-D-glucan testing and peritoneal fungal cultures were both negative. Empirical micafungin, an echinocandin, was administered for eight days before liposomal amphotericin B was initiated, during which time progressive fungal angioinvasion led to irreversible multi-organ failure and death. Histopathology confirmed broad, aseptate, right-angle branching hyphae invading gastric and splenic vessels, whereas no fungal elements were detected at the primary sigmoid lesion, supporting a predominantly hematogenous dissemination pathway without primary intestinal fungal colonization – an unprecedented finding that fundamentally distinguishes this case from classic primary gastrointestinal mucormycosis. These histopathological findings are biologically consistent with the CotH3 (CotH family protein 3) – GRP78 (glucose- regulated protein 78 kDa) host–fungal virulence axis, which represents a plausible mechanistic explanation for multi-organ angioinvasion observed in this patient.

**Conclusion:**

This case delivers three critical clinical takeaways. First, even brief stress-dose glucocorticoids may induce sufficient transient immune paralysis to permit life-threatening hematogenous mucormycosis in patients without chronic underlying immunosuppression, acting as one potential contributing factor alongside sepsis-related critical-illness immune dysfunction. Second, empirical carbapenem plus echinocandin regimens, commonly used in ICUs, lack activity against *Mucorales*; prolonged reliance on this combination can result in irreversible fatal visceral injury. Third, negative peritoneal fungal cultures and serum 1,3-β-D-glucan do not exclude intra-abdominal mucormycosis; early re-laparotomy for histopathological biopsy is essential in refractory cases.

## Highlights

1. This is the first reported fatal case of concurrent gastric perforation and splenic mucormycotic infarction in a completely immunocompetent adult with no chronic immunosuppressive comorbidities.
2. A 3-day stress-dose hydrocortisone regimen, together with septic-related immune paralysis following colonic perforation, may have resulted in the development of invasive mucormycosis – a potential contributing factor seldom documented in previous case series.
3. Absence of fungal hyphae at the primary sigmoid lesion favors a hematogenous route, an unprecedented finding that fundamentally differs from classic primary gastrointestinal mucormycosis.
4. Prolonged micafungin monotherapy led to irreversible multi-organ damage before targeted liposomal amphotericin B was administered, highlighting a critical, underrecognized therapeutic pitfall in postoperative intra-abdominal sepsis.

## Introduction

Mucormycosis is an aggressive angioinvasive infection caused by ubiquitous *Mucorales* fungi, including *Rhizopus*, *Mucor*, and *Lichtheimia*. Infection typically occurs through inhalation of spores, ingestion of contaminated food, or traumatic inoculation (Kontoyiannis and Walsh, 2026). Over recent decades, the global incidence has risen alongside expanding populations of immunocompromised patients and improved diagnostic capabilities (Imbert et al., 2023).

Classical risk factors include uncontrolled diabetes, hematological malignancies, solid organ transplantation, prolonged neutropenia, iron overload, and major trauma (Roden et al., 2005). These impair neutrophil/macrophage function while upregulating endothelial GRP78, a receptor for *Mucorales* adhesion (Alqarihi et al., 2020). Transient immune paralysis from septic shock, abdominal surgery, or short-course glucocorticoids also predisposes immunocompetent patients – an underrecognized risk group (Walsh et al., 2019; Hoffman et al., 2025).

Gastrointestinal mucormycosis accounts for only 7% of cases but has high mortality with perforation or dissemination (Roden et al., 2005). Primary gastrointestinal mucormycosis arises from ingested spores colonising injured mucosa, often with vague symptoms that delay diagnosis (Lee et al., 2014). Secondary abdominal mucormycosis following colonic perforation is less documented. Sepsis-related immunosuppression and glucocorticoids create a permissive environment for fungal angioinvasion, while peritoneal cultures and serum 1,3-β-D-glucan frequently yield false-negative results (Spallone et al., 2022).

Critically, no published case to date has documented concurrent gastric perforation and splenic infarction due to hematogenous *Mucorales* dissemination in a previously healthy adult, with the primary colonic lesion remaining fungus-free. All previously reported dual-visceral mucormycosis cases occurred in patients with chronic underlying immunosuppressive conditions. Furthermore, prior intestinal perforation-associated mucormycosis uniformly shows fungal infiltration at the primary bowel site, a feature notably absent in our patient.

A small number of published cases have described concurrent gastric and splenic mucormycosis secondary to gastrointestinal perforation or septic shock; however, nearly all of those patients harboured chronic immunosuppressive conditions including diabetes mellitus, hematological malignancy, or long-term immunosuppressive therapy. To our knowledge, none have reported hematogenous-predominant dissemination with complete absence of fungal elements within the primary intestinal perforation site in an otherwise immunocompetent host exposed only to short-course stress-dose glucocorticoids. Comparative details of these historical cases are summarized in **Table 1**.

**Table 1:** Comparative summary of published cases with gastric/splenic mucormycosis complicated by visceral perforation or infarction (2013–2025).

Source	Age	Baseline immunocompromising condition	Primary trigger of mucormycosis	Glucocorticoid exposure	Fungal-involved organs	Initial antifungal agent	Clinical outcome
Present case	55	None (no DM, malignancy, transplant, or chronic immunosuppression)	Spontaneous sigmoid colon perforation + septic shock	3 days of stress-dose hydrocortisone	Stomach (6.0×8.0 cm full-thickness rupture), Spleen	Micafungin	Death
Yang and Chen (2025)	73	None	Intestinal perforation, post-surgical recurrent GI bleeding	Not reported	Gastrointestinal tract	Amphotericin B	Death (family withdrew treatment)
AlBassam et al. (2025)	19	B-cell ALL, active chemotherapy	Neutropenic colitis	Chemotherapy-related immunosuppression (not steroid)	Ileocecal perforation	Amphotericin B	Survived (relapsed due to non-compliance, then retreated)
da Silveira et al. (2024)	56	Acute myeloid leukemia	Neutropenia, fungal disease	Not reported	Splenic infarction, gastric fistula, brain abscess	Broad-spectrum antifungal (not specified)	Death (postoperative day 2)
Kumta et al. (2024)	70s	None (immunocompetent)	Bowel perforation, post-laparotomy surgical wound infection	Not reported	Abdominal wall (surgical site)	None initially; debridement only	Death
Ralaizanaka et al. (2022)	75	Type 2 diabetes mellitus	Diabetes, acute peritonitis, hemorrhagic shock	Not reported	Multiple gastric ulcers, rectal perforation	Liposomal amphotericin B	Survived
Huang et al. (2021), Case 1	16	None (immunocompetent)	GI bleeding, gastric ulcer perforation	Not reported	Gastric ulcer perforation	Not specified (pathology confirmed GIM)	Survived
Huang et al. (2021), Case 2	33	None (immunocompetent)	Leg sprain, critical illness, hematemesis	Not reported	Gastric wall necrosis (total gastrectomy)	Anti-infective + antifungal (not specified)	Survived
Singh et al. (2021)	48	None (immunocompetent)	COVID-19, perforation peritonitis	Not reported (COVID-19 treatment not detailed)	Colonic perforation (sigmoid colectomy)	Injectable antifungal (not specified)	Survived
Termos et al. (2018)	52	Diabetes, hypertension, CKD on dialysis, heroin abuse	Chronic metabolic and renal dysfunction	Not reported	Total gastric necrosis, gastric perforation	Liposomal amphotericin B	Death (postoperative day 21)
Orosey et al. (2016)	44	None (immunocompetent); hypertension; HbA1c 11.9% (undiagnosed diabetes)	Abdominal distention, cecal perforation	No	Cecal perforation	IV amphotericin B	Survived
Enani et al. (2014)	54	Diabetes, hypertension, IHD, CKD, CHF	Gastric perforation, spleen infarction	Not reported	Gastric perforation, splenic infarction	None (not suspected preoperatively; no fungal culture)	Death
Kulkarni and Thakur (2015)	50	Diabetes, chronic alcoholism	Perforative peritonitis, gastric ulcer perforation	Not reported	Gastric body perforation (4×4 cm)	Not specified	Death
Kalva et al. (2013)	62	Wegener’s granulomatosis, immunosuppressed (Rituximab, Cyclophosphamide)	Immunosuppression, pulmonary aspergillosis, bowel perforation	Prednisone (long-term)	Jejunum and ileum, multiple full-thickness perforations	Voriconazole + Caspofungin (for aspergillosis; no anti-Mucor activity)	Death (within 24 hours)

ALL, acute lymphoblastic leukemia; CHF, congestive heart failure; CKD, chronic kidney disease; DM, diabetes mellitus; GI, gastrointestinal; GIM, gastrointestinal mucormycosis; HbA1c, glycated hemoglobin; IHD, ischemic heart disease; IV, intravenous. Among all 12 reviewed published cases, the current case is the only one with dual gastric and splenic mucormycosis occurring in a fully immunocompetent host without chronic underlying immunosuppressive disorders, driven solely by short-term stress glucocorticoids and hematogenous spread.

We report this unprecedented fatal case of synchronous gastric rupture and total splenic infarction in a fully immunocompetent male following spontaneous sigmoid perforation and short-course stress-dose glucocorticoids therapy for septic shock. We delineate the distinctive host-pathogen interplay, highlight diagnostic pitfalls, compare our case with published literature, and offer actionable clinical guidance for intensivists and mycologists.

## Case presentation

### Patient information and initial presentation

A 55-year-old married male civil servant, with no significant history except prostate surgery, presented on 11 September 2025 with two-day left abdominal pain, altered mental status, and high fever (39.0 °C). He had no nausea, vomiting, cough, dyspnea, hematemesis, or flatus passage, and had no constipation. On admission, the patient was in septic shock: temperature 38.5 °C, pulse 123 beats/min, respiratory rate 30 breaths/min, and blood pressure 76/45 mmHg. He was agitated with cold extremities and cyanotic nail beds. Abdominal examination revealed mild tenderness in the periumbilical and left lower quadrant without obvious peritoneal signs; bowel sounds were absent.

### Clinical findings on admission

Laboratory tests showed: white blood cell count 5.91 × 10^9^/L (neutrophils 94.4%), platelet count 42 × 10^9^/L, lactate 5.4 mmol/L, procalcitonin 78.34 ng/mL, C-reactive protein 285 mg/L, creatinine 267 μmol/L, alanine aminotransferase 2181 U/L, aspartate aminotransferase 10,877 U/L, and total bilirubin 69.5 μmol/L. Abdominal computed tomography (CT) revealed a small amount of free intraperitoneal gas, bilateral lower lobe pneumonia with small pleural effusions, and ascites. Diagnostic abdominal paracentesis yielded feculent fluid.

### Timeline

The key clinical events, diagnostic procedures, and therapeutic interventions are summarized in **Table 2**.

**Table 2:** Key clinical events timeline of the patient.

Time point	Core clinical event	Critical intervention	Key diagnostic findings
Admission	Spontaneous sigmoid colon perforation, diffuse peritonitis, septic shock	Emergency sigmoidectomy + abdominal irrigation	CT: free intraperitoneal gas, fecal ascites
POD 0–3	Refractory septic shock	Hydrocortisone 200 mg/d for 3 days	Progressive thrombocytopenia, renal impairment
POD 5	Persistent fever, abdominal sepsis	Micafungin added empirically	Drainage culture: Proteus mirabilis, no fungi; negative serum (1,3)-β-D-glucan test
POD 10	Shock deterioration, massive gastric perforation, splenic infarction	Re-laparotomy, total gastrectomy + splenectomy; surgical specimens submitted for histopathology	Intraoperative findings: gastric perforation, splenic necrosis
POD 13	Histopathological confirmation of mucormycosis; irreversible multi-organ failure	Liposomal amphotericin B initiated immediately upon diagnosis; supportive care	Histopathology: *Mucorales* angioinvasion in gastric and splenic tissue; refractory lactic acidosis, anuria
Outcome	Fatal circulatory collapse	—	—

POD, postoperative day; CT, computed tomography.

### Diagnostic workup and treatment (POD 0–3)

Emergency exploratory laparotomy was performed with a diagnosis of diffuse peritonitis due to colonic perforation. Intraoperatively, a 0.8 × 0.8 cm perforation was identified on the lateral wall of the sigmoid colon, approximately 2 cm distal to the rectosigmoid junction. No other perforations were found in the stomach or remaining bowel. Partial sigmoid colectomy with colostomy and abdominal irrigation and drainage were performed.

Postoperatively, the patient remained hemodynamically unstable with refractory hypotension (50–60/30–40 mmHg), anuria, and progressive thrombocytopenia. He received fluid resuscitation, norepinephrine, and continuous intravenous hydrocortisone at a daily dose of 200 mg for 3 days for septic shock. On postoperative day 2, he was transferred to the intensive care unit, where he required mechanical ventilation and continuous renal replacement therapy (CRRT). Empirical antibiotic therapy with meropenem was administered, along with ulinastatin for anti-inflammatory therapy and immunoenhancement.

### Diagnostic workup and treatment (POD 5–10)

On POD 4, mental status and hemodynamics improved, and he was extubated. However, on POD 5, fever and tachypnoea recurred. Drainage fluid grew Proteus mirabilis (no fungi); serum 1,3-β-D-glucan was negative. Antimicrobials were adjusted to meropenem plus empirical micafungin based on susceptibility and fungal risk. Micafungin, an echinocandin agent, was selected as empirical antifungal therapy to cover suspected intra-abdominal invasive candidiasis, a common complication following fecal peritonitis. At that time, there were no clinical, microbiological or imaging signals pointing toward mucormycosis.

Despite this regimen, the patient remained febrile and the peripheral white blood cell count continued to rise. A repeat CT scan on postoperative day 8 demonstrated bilateral lower lobe pneumonia with pleural effusion, thickened small bowel walls, and persistent ascites.

### Diagnostic assessment – second surgery and histopathology

On postoperative day 10, the patient developed refractory septic shock with large amounts of dark brown fluid draining from the right upper abdominal and pelvic drains, raising concern for an enteric fistula. An emergency second laparotomy was performed. Intraoperative findings included a small amount of purulent intra-abdominal fluid, significant bowel wall oedema and inflammation, and a large loculated collection of purulent digestive fluid within the lesser omental sac. A 6.0 × 8.0 cm perforation was identified on the posterior wall of the stomach (**Figure 1A**). The spleen appeared ischemic and necrotic (**Figure 1B**). Total gastrectomy with splenectomy, jejunostomy, and oesophagostomy were performed.

**Figure 1:**
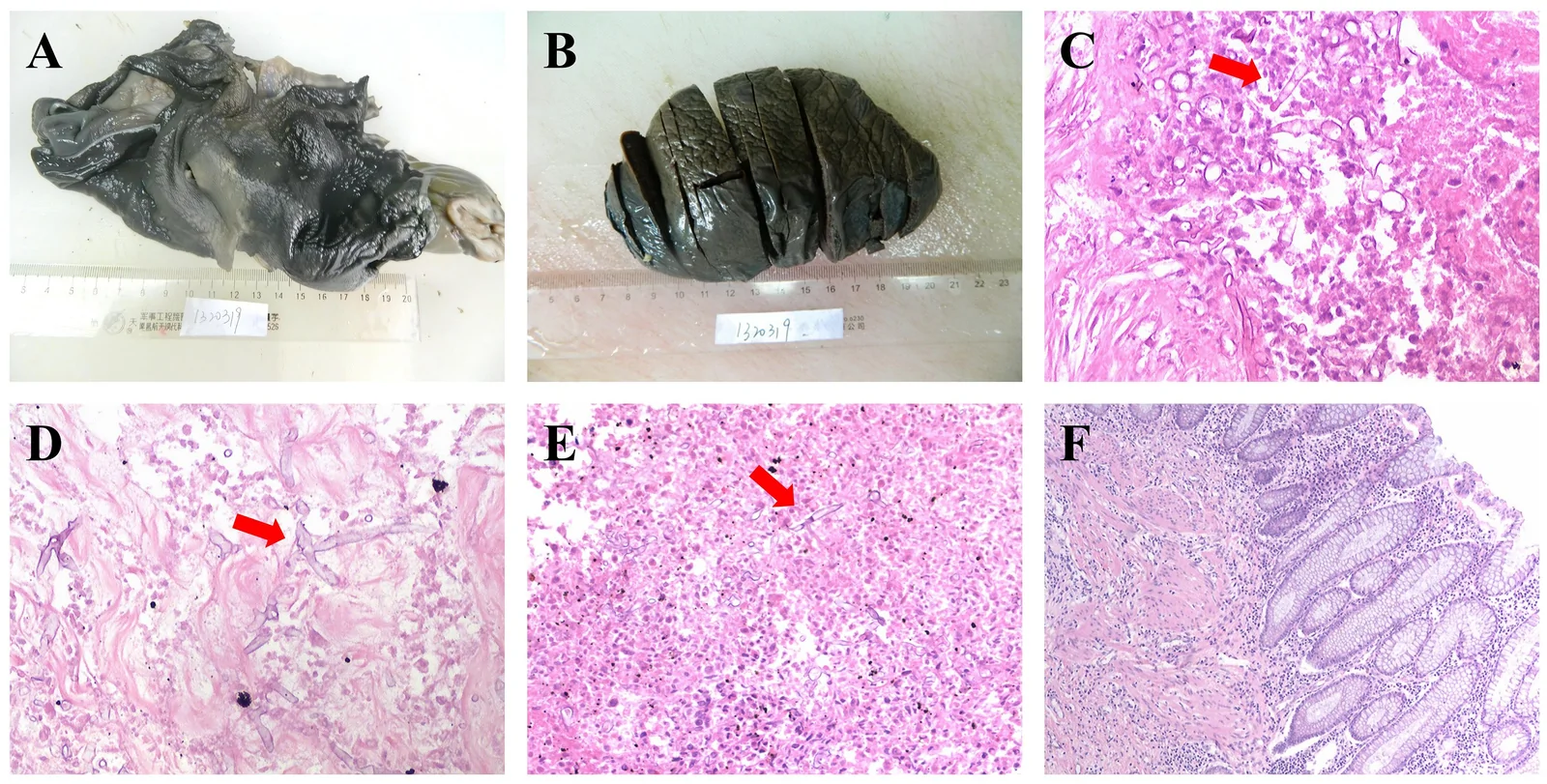
Intraoperative gross specimens and histopathology of secondary abdominal mucormycosis complicated by concurrent gastric perforation and splenic infarction. **(A)** Resected stomach gross specimen. Everted posterior wall perforation; black mucosa from necrotic infarction. **(B)** Sectioned splenic gross specimen. Diffuse ischemic necrosis caused by fungal angioinvasion; sampling incisions visible. **(C)** Gastric wall, H&E staining, ×400 magnification. Broad, aseptate, right-angle *Mucorales* hyphae infiltrate vessels (red arrow), resulting in intraluminal thrombosis and hemorrhagic infarction. **(D)** Gastric mucosa, H&E staining, ×400 magnification. Dense Mucorales hyphae in necrotic tissue (red arrow), with acute inflammation and tissue damage. **(E)** Splenic tissue, H&E staining, ×200 magnification. Vascular invasion by *Mucorales* hyphae (red arrow), occlusive thrombosis and widespread hemorrhagic infarction with mixed inflammatory infiltrates. **(F)** Perforated sigmoid colon, H&E staining, ×100 magnification. Mural hemorrhage, infarction and thrombosis; no fungal hyphae detected, ruling out primary colonic mucormycosis.

Histopathological findings (**Figures 1C–F**):

Stomach: Full-thickness hemorrhagic infarction with extensive infiltration by broad, aseptate or pauciseptate, ribbon-like hyphae with right-angle branching, characteristic of *Mucorales*. Fungal elements invaded blood vessel walls with associated thrombosis. The gastric mucosa showed numerous fungal hyphae. The serosal surface contained bacterial colonies and gastrointestinal contents. These findings were consistent with gastric mucormycosis, hemorrhagic infarction, and acute perforation.

Spleen: Acute splenitis with mucormycosis infection and infarction.

Sigmoid colon: Perforation site with hemorrhagic infarction, thrombosis, and acute pericolitis. No fungal elements were identified in the colonic specimen. Multiple-level serial sections were generated from the sigmoid perforation margin and adjacent injured bowel wall. All hematoxylin-eosin-stained slides were reviewed by two pathologists, and no *Mucorales* hyphae were detected across all examined sections, excluding sampling-caused false-negative results.

### Differential diagnosis

Ischemic gastritis and splenic infarction secondary to bacterial sepsis. Histological examination shows only inflammatory infiltration, without transmural fungal invasion or pathognomonic broad, right-angle branching *Mucorales* hyphae; diffuse vascular thrombosis induced by fungi is absent.

Invasive aspergillosis (Tomazin and Matos, 2026). Pathology demonstrates septate, dichotomously branching hyphae. Serum galactomannan testing is frequently positive, and echinocandins exert partial therapeutic activity against Aspergillus, which contradicts the treatment failure observed in our patient.

Primary stress-induced gastric perforation. This entity only presents with superficial mucosal erosion rather than full-thickness fungal vascular invasion, and cannot account for concurrent splenic infarction.

Primary gastrointestinal mucormycosis. This entity typically occurs in patients with diabetes or malnutrition, with fungi confined to the intestinal lesion. By contrast, no fungal hyphae were detected in our patient’s sigmoid colon; gastric and splenic *Mucorales* infection arose secondarily via hematogenous spread.

### Therapeutic intervention – antifungal therapy

Empiric micafungin was initiated on POD 5 for persistent abdominal sepsis. Re-laparotomy with total gastrectomy and splenectomy was performed on POD 10, and surgical specimens were submitted immediately for histopathological examination. Definitive histopathological confirmation of mucormycosis became available on POD 13, at which point liposomal amphotericin B was immediately commenced and micafungin discontinued. However, despite maximal intensive care, the patient developed refractory shock and systemic capillary leak syndrome, and died on POD 13, shortly after the diagnosis was established.

## Discussion

### Unique clinical phenotype and pathogenic mechanism

We report a fatal case of synchronous gastric perforation and splenic infarction secondary to mucormycosis arising after spontaneous sigmoid colon perforation. To our knowledge, this dual-visceral angioinfarction phenotype has seldom been documented in prior publications.

Mucormycosis, an opportunistic infection caused by fungi of the order *Mucorales*, is characterized by prominent angioinvasion leading to vascular occlusion and tissue necrosis. The pathogenic success of *Mucorales* relies on a multifaceted virulence arsenal, including high-affinity iron scavenging, the ricin-like toxin mucoricin, and cell wall remodeling that masks pathogen-associated molecular patterns (Alanazi et al., 2026). Among these, the CotH3–GRP78 (CotH family protein 3–glucose-regulated protein 78 kDa) virulence axis represents a plausible molecular pathway that could explain multi-organ injury observed in this patient (Alqarihi et al., 2020; Alqarihi et al., 2023). Hyphae of *Mucorales* express surface CotH3 adhesins that selectively bind endothelial GRP78 receptors. Stress-induced transient hyperglycemia, lactic acidosis from severe sepsis, and short-term exogenous glucocorticoids work in concert to upregulate endothelial GRP78 expression in gastric and splenic vasculature, enhancing fungal attachment, arterial thrombosis, and full-thickness tissue destruction. These histopathological findings are biologically consistent with this molecular cascade, which may underlie the synchronous gastric rupture and diffuse splenic necrosis seen in our case.

The septic milieu in our patient – lactic acidosis (5.4 mmol/L) and stress-induced hyperglycemia – likely synergized with glucocorticoids to maximize GRP78 upregulation. Acidic pH, elevated glucose, and glucocorticoids independently upregulate endothelial GRP78 (Alqarihi et al., 2020; Alqarihi et al., 2023), creating a transient “vascular adhesion window” that may suffice to convert quiescent endothelium into a permissive surface for CotH3-mediated attachment. Unlike chronic immunosuppression, acute metabolic dysregulation plus brief steroids can achieve a similar endpoint within days, aligning with data showing GRP78 upregulation is rapid and reversible (Alqarihi et al., 2023).

The preferential gastric and splenic vasculature involvement, with sparing of other organs, merits consideration. Classic gastrointestinal mucormycosis follows a luminal route: spores germinate on damaged mucosa, invade submucosally, and spread contiguously. Our sterile sigmoid lesion favors hematogenous seeding of the stomach and spleen. We cannot fully rule out an unrecognized minor gastrointestinal portal of entry that was not surgically sampled, although no fungal elements were detected within the resected sigmoid perforation specimen. Why these two organs? GRP78 is constitutively higher in splenic sinusoidal and gastric mucosal endothelium than in colonic or hepatic endothelium (Alqarihi et al., 2020; Alqarihi et al., 2023). Sepsis-induced global GRP78 upregulation may amplify these baseline differences, creating susceptible vascular beds. This is supported by extensive vascular thrombosis in both organs, a hallmark of CotH3-GRP78-mediated angioinvasion. Our case suggests endothelial receptor heterogeneity, not random embolization, may govern hematogenous spread.

Several factors jointly facilitated mucormycosis. First, sigmoid perforation caused fecal peritonitis, septic shock, and critical illness-associated immune paralysis. Second, hydrocortisone (200 mg/d for 3 days), though guideline-concordant, impairs neutrophil/macrophage function; glucocorticoid-induced immunosuppression disrupts mucosal barriers and attenuates antifungal defenses (Murugavel and Ramakrishnan, 2026). While sepsis-associated immune paralysis and surgical stress are major contributing factors, the superimposed short-term glucocorticoid course may have further amplified this transient immunosuppressive state. Third, although iron studies were not measured, the stress-induced hyperglycemia and acidosis represent a metabolic milieu known to increase bioavailable free iron, which fuels *Mucorales* growth.

Besides the CotH3-GRP78 adhesion cascade, alternative pathogenic mechanisms may contribute to endothelial damage in mucormycosis. These include cytotoxic injury induced by the ricin-like toxin mucoricin, siderophore-mediated iron acquisition facilitating fungal proliferation, integrin-dependent host-fungal binding, and sepsis-driven impairment of neutrophil-mediated antifungal defense. Given the retrospective nature of this case, we cannot quantify the relative contribution of each pathway to the observed gastric and splenic vascular injury (Ibrahim et al., 2012; Alqarihi et al., 2020; Soliman et al., 2021).

### Literature comparison: What makes this case different?

Comparative analysis of the 12 histologically confirmed cases retrieved from PubMed (2013–2025) (**Table 1**) highlights three unique features of our report.

First, this case represents a rare subtype of transiently immunocompromised mucormycosis with a distinctly minimal risk profile. In all prior cases with concurrent gastric and splenic angioinvasion (Enani et al., 2014; da Silveira et al., 2024), patients harboured permanent chronic immunosuppressive conditions, hematological malignancy, long-standing diabetes, advanced cardiorenal disease, or sustained anti-rejection therapy. Even the previously reported “immunocompetent” cases (Huang et al., 2021; Singh et al., 2021; Yang and Chen, 2025) lacked explicit documentation of glucocorticoid exposure as a precipitant. By sharp contrast, our patient had none of these classic predispositions; the only synergistic drivers were three consecutive days of stress hydrocortisone for septic shock, superimposed upon transient immune paralysis from fecal peritonitis. This near-absent baseline risk profile has scarcely been recorded in combined gastric-splenic mucormycosis cohorts.

Second, we establish a novel secondary hematogenous pathogenic trajectory, distinct from classic primary gastrointestinal mucormycosis. In primary gastrointestinal mucormycosis, ingested spores colonize the locally injured intestinal wall. However, pathological examination of our sigmoid perforation specimen definitively excluded fungal infiltration at the primary colonic site. This confirms a unique disease cascade: colonic perforation-induced sepsis plus brief glucocorticoid exposure jointly impaired host antifungal immunity, enabling hematogenous *Mucorales* dissemination to the stomach and spleen, causing synchronous full-thickness gastric rupture and total splenic angioinfarction. Such a secondary bloodstream spread pattern, with a sterile primary colonic lesion, has not been previously described in comparable visceral perforation cases.

Third, this report exposes underrecognized diagnostic and therapeutic pitfalls. Early empirical therapy with meropenem plus micafungin, a standard ICU regimen for abdominal sepsis, was instituted. However, routine microbiological tests returned false-negative results, and the patient deteriorated until histopathology confirmed mucormycosis. These pitfalls are discussed in detail in the following section.

Concurrently, routine peritoneal fungal cultures and serum 1,3- β -D-glucan yielded false-negative results, delaying definitive diagnosis until repeat laparotomy and histopathology. The key clinical warning for surgeons and intensivists is this: in postoperative patients with refractory intra-abdominal sepsis that fails to respond to antibacterial echinocandin coverage, mucormycosis must be prioritized in the differential diagnosis, even in the absence of traditional long-term immunocompromising comorbidities.

### Diagnostic and therapeutic pitfalls

This case fully illustrates multiple diagnostic challenges of postoperative secondary intra-abdominal mucormycosis. Consistent with previous reports, peritoneal fungal cultures are positive in fewer than half of intra-abdominal mold infections, and blood cultures offer even poorer diagnostic performance (Skiada et al., 2018). The serum 1,3- β -D-glucan test cannot rule out *Mucorales*, as these fungi contain minimal cell-wall glucan; definitive diagnosis thus depends on surgical histopathology identifying pathognomonic broad, aseptate, right-angle branching invasive hyphae (Cornely et al., 2019).

Molecular tools including *Mucorales*-specific quantitative polymerase chain reaction (qPCR) and metagenomic next-generation sequencing (mNGS) enable early detection of fungal DNA before culture positivity. mNGS is particularly valuable as a culture-independent method that directly detects microbial cell-free DNA in clinical specimens, with reported sensitivity superior to conventional methods for *Mucorales* (Lackner et al., 2021). Early mNGS testing of peritoneal fluid or tissue should be strongly considered when intra-abdominal mucormycosis is suspected, especially in critically ill surgical patients where repeated biopsies carry high risk. In our case, although mNGS was offered to the family, it was declined, precluding timely molecular confirmation. Broader acceptance and earlier application of this tool could help shorten diagnostic delays and improve outcomes in similar settings.

From a therapeutic standpoint, radical surgical debridement combined with prompt, targeted antifungal therapy constitutes the cornerstone of mucormycosis management. At present, universal standardized susceptibility breakpoints for *Mucorales* have not been established, restricting the clinical interpretive value of minimum inhibitory concentration data. *In vitro* experiments, preclinical animal models, and real-world clinical studies uniformly demonstrate that echinocandins, flucytosine, fluconazole, itraconazole, and voriconazole lack meaningful activity against *Mucorales*. In contrast, amphotericin B formulations, isavuconazole, and posaconazole display proven *in vivo* and clinical efficacy. Liposomal amphotericin B is the gold-standard first-line agent, while isavuconazole and posaconazole are preferred for sequential maintenance treatment (Cornely et al., 2019; Hoffman et al., 2025).

Our patient’s clinical course directly corroborates this established susceptibility profile. Micafungin, an echinocandin, failed to halt progressive fungal injury and persistent fever. Critically, **Table 2** highlights a key therapeutic delay: micafungin monotherapy was initiated on POD 5, yet liposomal amphotericin B was not administered until POD 13, creating an 8-day window of ineffective antifungal coverage. This prolonged lag in appropriate targeted therapy enabled unimpeded *Mucorales* angioinvasion and widespread visceral necrosis, which culminated in irreversible multi-organ failure and death. **Table 2** also demonstrates a notable chronological association between glucocorticoid exposure and infectious deterioration: hydrocortisone was administered daily from POD 0 to POD 3, and refractory fever and abdominal sepsis emerged as early as POD 5 shortly after glucocorticoid cessation. This temporal relationship suggests that transient glucocorticoid-related immune paralysis may have favored occult fungal dissemination.

### Strengths, limitations, and clinical implications

The major strength of this report is the histopathological confirmation of synchronous gastric and splenic mucormycosis in a fully immunocompetent adult without chronic comorbidities, with a sterile primary colonic lesion – a phenotype not previously documented in the literature.

However, this case report has several limitations. First, as a single retrospective observation without a control cohort, quantitative comparative analysis is not feasible. Second, although histopathology confirmed *Mucorales* angioinvasion, fungal cultures of drainage fluid remained negative, preventing species-level identification via matrix-assisted laser desorption/ionization time-of-flight mass spectrometry (MALDI-TOF) or tissue sequencing. mNGS, a highly sensitive culture-independent molecular tool, was offered to the family but declined, precluding early pathogen detection. Third, the patient died shortly after reoperation and definitive diagnosis; therefore long-term response to antifungal therapy could not be assessed. Furthermore, we could not experimentally verify activation of the CotH3-GRP78 virulence axis in this patient’s surgical specimens. Although this molecular pathway is well supported by *in vitro* and animal experimental data, we did not perform immunohistochemical staining or molecular testing for fungal CotH3 or host endothelial GRP78. We can only state that our histopathological findings are biologically compatible with this proposed mechanism, and future case studies should consider applying tissue-based molecular or immunohistochemical assays to evaluate this pathway in clinical samples. Fourth, iron-related biomarkers were not assessed. Although siderophore-dependent iron acquisition is a recognized pre-clinical virulence mechanism of *Mucorales*, the ECMM-MSG guideline does not recommend routine iron-profile testing for critically-ill surgical sepsis patients (Cornely et al., 2019). Ferritin results are confounded by severe inflammation, no validated diagnostic cut-offs exist, and *Mucorales*-specific siderophore assays are experimentally-only and locally unavailable. Thus, the hypothesized metabolic-iron link cannot be verified retrospectively; iron-related biomarkers are suggested for future prospective studies. Fifth, findings from a single case are not generalizable, and larger multicenter studies are needed to validate our observations in this specific clinical context. The absence of species-level identification also limits further analysis of strain-specific virulence characteristics. These caveats should be considered when extrapolating the clinical takeaways from this single fatal case.

Despite these limitations, three key clinical warnings emerge for intensivists and surgeons:

1. Even short-term guideline-recommended stress-dose glucocorticoids, as brief as three days, may precipitate fatal dual-visceral mucormycosis in patients without classic risk factors.
2. Carbapenem plus echinocandin, a standard empirical regimen for abdominal sepsis, has no activity against *Mucorales*; reliance on this combination can dangerously delay effective therapy.
3. Negative peritoneal fungal cultures and serum 1,3-β-D-glucan do not exclude intra-abdominal mucormycosis; reoperation for histopathological biopsy should be pursued early in refractory cases.

When critically ill surgical patients present with refractory intra-abdominal sepsis unresponsive to antibacterial and echinocandin regimens, clinicians must place mucormycosis high on the differential diagnosis list. Early re-laparotomy for histologic biopsy and prompt initiation of liposomal amphotericin B are life-saving interventions to lower mortality. In summary, this case underscores that even brief, guideline-recommended glucocorticoid courses may precipitate fatal mucormycosis in patients without traditional risk factors. For intensivists and surgeons, maintaining a high index of suspicion, obtaining early histopathological confirmation, and promptly initiating liposomal amphotericin B are paramount to improving outcomes in refractory postoperative intra-abdominal sepsis.

## Patient perspective

The patient remained sedated under mechanical ventilation from postoperative day 2 until death on postoperative day 13, so no subjective treatment experience could be obtained from the patient himself. Written informed consent for publication of clinical data, intraoperative images and histopathological slides was fully acquired from the patient’s legal next of kin.

## Data Availability

The original contributions presented in the study are included in the article/supplementary material. Further inquiries can be directed to the corresponding author.
